# 

**Published:** 1915-11

**Authors:** 


PUBLISHED MONTHLY. ATLANTA SUBSCRIPTION $1.00

### JOURNAL-RECORD OF MEDICINE

LIBRARY
DEC. 16. 1915
SURGEON GENERAL'S OFFICE

Successor to { Atlanta Medical and Surgical Journal, Established 1855. and Southern Medical Record, Established 1870. } 62nd Year

Vol. LXII ATLANTA, GA., NOVEMBER, 1915 No. 8

